# The Value of the Microscope in Diagnosis and Treatment of Tuberculosis

**Published:** 1892-01

**Authors:** J. Samuel Price

**Affiliations:** County Health Officer, Jefferson county, Texas


For Daniel's Texas Medical Journal.

### THE VALUE OF THE MICROSCOPE AS AN AID IN THE DIAGNOSIS AND TREATMENT OF INCIPIENT AND ADVANCED PULMONARY TUBERCULOSIS.

BY J. SAMUEL PRICE, M. D.,
County Health Officer, Jefferson county, Texas.

ONLY A FEW YEARS have elapsed since that distinguished gentleman, Koch, discovered, after many years of careful study and arduous research, the true etiological factor of that most baneful disease, tuberculosis. His teachings, like all other profound students of science, are so clear that a tyro in medicine, or a layman, cannot but grasp, at once, the vast importance of his discovery as bearing on practical and preventive medicine. Not unlike many other discoveries in the scientific world, however, which at first arouses a lively interest in our minds, we are prone to forget the incentive that stimulated this great and good man in his magnanimous work in behalf of his fellowman, and permit his memory to drift into partial oblivion; but yet we can not forget the beneficence of his simple but invaluable truths.

It is not the object of this paper to tire the reader by an intricate review of the bacteriological research that led to the discovery of the bacillus tuberculosis, but preferably to insist upon the paramount importance of ascertaining the presence or absence of this germ in all cases coming to us that are in the least suspicious whatever from diathesis, or some predisposition not diathetic, which renders it possible for the tubercular process to become ingrafted. Such cases come to us daily, and notwithstanding our advanced knowledge in the art of physical diagnosis, we often send our patient away with the gratifying assurance that it is only a slight bronchial trouble which we believe readily amenable to treatment, while in fact we are having to do with a genuine case of incipient pulmonary tuberculosis, which we stumble over, either by a hurried examination or, perchance, absolute ignorance of those signs that at least warrant us in suspecting consumption, when if we had resorted to the simple expedient of examining the sputum the microscope would have revealed to us not only our ignorance, but the significance of the signs elicited.

Professors in our leading medical colleges are beginning to recognize and insist upon the inportance of the acquirement by the student of at least that amount of knowledge that will enable him to mount and stain the sputa of all patients, the physical signs of whom render their cases in the least doubtful; and this knowledge, though meagre in the microscopical and bacteriological world, when attained by the busy practitioner places him in an attitude where he can act in the best interest of his anxious patient; and if there be any remedial measures, whether medicinal, dietetic or climatic, that might be of benefit to those thus afflicted, they will be accorded the advantage of early treatment, and will not live to deride the wise conclusion of their physician when in the advanced stage of tuberculosis. Such egregious blunders are very reprehensible, in view of our present knowledge of arresting the insidious invasion of this dread disease, which is daily robbing our homes of our friends and loved ones.

Let us, after an instant, consider how we may not only make clear the presence of the bacillus tuberculosis, but by repeated examinations of the sputum from time to time, we will, by observing the increase or decrease in the number of bacilli in the successive fields examined, keep constantly familiar with the exact progress, whether favorable or otherwise, of our charge.

This, to any common-sense doctor, should be clear, and recommend itself to his armamentarium as an invaluable aid to him, if he presume to become responsible for the lives of those individuals who place their destiny in his hands. Believing, as we do, that there is no one agent known capable of arresting the tubercular process, it becomes our duty to cast around for those measures which science has demonstrated to be capable of benefiting such patients, and through our microscope we can keep constantly informed as to the benefit derived from the measures instituted.

Prof. Biggs, in a paper published in "Buck's Reference Hand-Book of the Medical Sciences," says: "The tubercle bacillus is the active cause of this disease, and is the only active cause. If introduced in large enough numbers this will produce the disease independently of any other influence, but under natural conditions, if no local or general depression of nutrition exists, the intensity of exposure to infection or the number of tubercle bacilli received at any given time, is insufficient to overcome the natural insusceptibility When, however, the natural resisting power to the disease is lessened from any cause, the dose of tubercle bacilli, that before proved insufficient, now results in the production of the disease. Further than this, the same conditions exist later on in the disease, and influence in the same manner the extension of the tubercular process. If the tissues possess a certain degree of vigor, and if the standard of nutrition is brought up to a certain level, they resist the multiplication of the tubercle bacilli and the extension of the tubercular process. This then becomes stationary or retrogressive. If, on the other hand, the vitality of the tissue is low, and the conditions are favorable for the development of the tubercle bacilli, they find little resistance to their multiplication and the disease rapidly extends. That this is exactly what occurs again and again, is shown clearly enough by the conditions found after death. In nearly fifty per cent. of the autopsies in hospital cases, there are found after death the evidence of a tubercular process in some part of the body that has existed some time in life. Probably not more than twenty per cent. of deaths result directly from this disease, and in the other thirty per cent., or more than half of the cases of tubercular disease remained strictly localized, and in many instances has long since become stationary or retrogressive. In most of these cases the individuals have probably never been conscious of the existence of any disease. But at some time in life, as the result of some depressing influences, conditions favorable to the development of the tubercle bacilli have been produced, the patients have been exposed to infection and a local tuberculosis has resulted. Later, the conditions have changed, the local or general state of nutrition has improved, and with this has come a corresponding increase in the resisting power of the tissues; they no longer afford a favorable soil for growth of the germs, and the disease ceases to extend."

We have dwelt somewhat at length on what at first sight might appear irrelevant to the title of this paper, but unless we keep clearly in our minds the fact that consumption is a curable disease, and that patients are daily recovering therefrom, we may, perhaps, deem the expenditure necessary for a first-class instrument, that much money placed in a toy that is used only by those enthusiasts in science who are mere "*library practitioners,*" known only for their fondness of airing their medical lore; but as we watch the appearance and disappearance of casts in the excrement of patients suffering from nephritis, so should we with equal interest observe the same process relating to the increase and decrease of the bacilli tuberculosis in the sputa of our consumptive charge. An instrument that has a magnifying power of three hundred diameters is sufficient for revealing the preseace of the bacillus tuberculosis, which we will remember as a "slender motionless rod, about five times as long as broad, seldom seen perfectly straight, generally curved, often bent upon itself, with rounded ends, and provided with spores which represent its permanent form."

We have found the following a good method of staining, and if given a fair trial, we believe it will recommend itself to workers in this field:

Put a small quantity of the sputum upon a cover-glass and spread it out *very thin*—guard against using too much. Dry thorougly and carefully over an alcohol lamp, being careful not to char it; next color it with magenta by putting two or three drops upon the glass and dry again carefully, in order to stain the bacilli and tissue. Then dip the glass in a solution of nitric acid and water, one part of the former to two parts of the latter, then again dip glass in pure water several times, each time holding the glass on a level to prevent washing the bacilli off; dry again, and by this means the tissue is bleached and the bacilli remain colored. Next put a thin layer of balsam on a slide and place the cover-glass upon it, pressing it down well, when it is ready for examination.

The magenta is an analine preparation containing one part magenta, five parts carbolic acid, ten parts of alcohol and one hundred parts water; it is of a violet color, or perhaps more of a red.

Bacilli remain colored and do not bleach as tissue does, because they are supposed to possess a great affinity for the coloring matter and seem to "fix" the dye.

